# Ectopia cordis associated with Cantrell's pentalogy

**DOI:** 10.4103/1817-1737.43084

**Published:** 2008

**Authors:** Basant Kumar, Chetan Sharma, Devendra D. Sinha, Sumanlata

**Affiliations:** *Department of Pediatric Surgery, Sir Padampat Mother and Child Health Institute (JayKayLon Hospital), SMS Medical College, Jaipur, Rajasthan, India*; 1 *Community Health Center, Jalalabad, Shahjahanpur, (UP), India*

**Keywords:** Abdominal wall defect, Cantrell's pentalogy, ectopia cordis

## Abstract

Cantrell's pentalogy with ectopia cordis is an extremely rare and lethal congenital anomaly, with a reported incidence of 1:100000 births in developed countries. We report a neonate who presented with ectopia cordis along with cleft lower sternum, upper abdominal wall defect, ectopic umbilicus, diaphragmatic defect, and interventricular septal defect. The neonate had respiratory distress with peripheral cyanosis and died because of acidosis and electrolyte imbalance before surgical intervention could be undertaken. We discuss the case and present a brief review of literature and of embryogenesis

Of all congenital anomalies, ectopia cordis, i.e., the presence of a live, beating heart outside the thorax, is perhaps the most distressing; it gives a grotesque appearance to neonate. This condition is extremely rare and is usually associated with Cantrell's pentalogy or a variant of Cantrell's pentalogy with sternal defect.[1] We discuss a case of ectopia cordis associated with Cantrell's pentalogy and present a brief review of the literature. We report this case because of its rarity.

## Case Report

**Figure 1:**
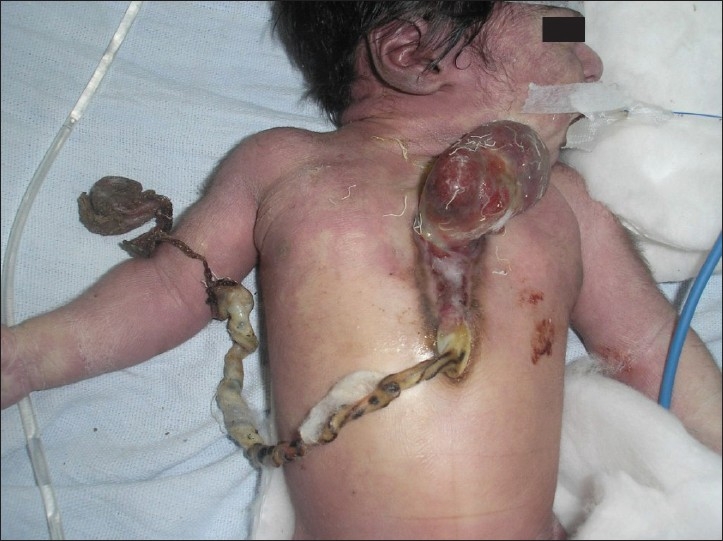
Photograph showing ectopia cordis and ectopic umbilicus with sternal and upper abdominal wall defect.

A 20-hour-old full-term male neonate presented to us with complaints of an externally visible, beating heart over the chest wall and difficulty in respiration. The neonate was delivered at home by a normal vaginal delivery and had a birth weight of 2.5 kg. He was the first issue of his 28-year-old mother. The antenatal history was unremarkable and there was no history of exposure to any unusual infection or drug. Antenatal ultrasonography had been done by a local practitioner but the deformity was not detected, probably due to lack of experience. There was no family history of any congenital abnormality. At the time of presentation, the neonate had peripheral cyanosis and there was history of difficulty in breathing and of vomiting since birth. The beating heart, covered with a serous membrane, was visible in the middle of the chest wall. The lower half of the sternum and the upper abdominal wall was deficient and was covered with a thin membrane. The umbilical cord was attached at the lower part of the abdominal wall defect near the epigastric region and the abdomen was scaphoid [Figure 1]. Other than this, no external deformity was found in any other part of the body. Chest movement on the left side was absent and no air entry could be made out on auscultation. X-ray of the chest revealed a left-sided diaphragmatic hernia with mediastinal shift. Echocardiography showed interventricular septal and pericardial defects. Blood gas analysis revealed severe respiratory acidosis and dyselectrolytemia. The neonate was resuscitated and put on mechanical ventilation (pressure-limited, time-cycled). Continuous nasogastric aspiration and broad spectrum antibiotics were started. Appropriate measures were taken to correct hypothermia and dehydration. We tried to correct the acidosis and electrolyte imbalance, but the neonate did not show any improvement either clinically or on blood gas analysis and expired before any surgical intervention could be undertaken. Autopsy was not done.

## Discussion

The pentalogy of Cantrell is an extremely rare congenital anomaly. The complexity of this syndrome is usually incompatible with life, so the exact incidence could not be found in the literature; however, an incidence of 1:100000 births, with a male preponderance (M : F = 2 : 1.2), has been described in literature in developed countries.[2] Cantrell, Haller, and Ravitch, in 1958, were the first to describe this syndrome, which is characterized by a midline supraumbilical abdominal wall defect, a defect of the lower sternum, a deficiency of the anterior diaphragm, a defect in the diaphragmatic pericardium, and congenital intracardiac defects.[1–3] Displacement or eventration of the heart through the abdominothoracic wall defect is called ectopia cordis. Depending on the location of the protruding heart and on the extent of the body wall defect, ectopia cordis may be grouped into cervical, thoracic, thoracoabdominal, or abdominal types.[3] The heart was uncovered in 41%, covered with a serous membrane in 31%, and covered with skin in 27% of reported cases.[3] In thoracoabdominal ectopia, as in our case, the body wall usually remains unclosed up to the umbilicus. The diaphragm has a V-shaped hiatus. The anterior and inferior portion of the pericardium may be absent. The heart itself may be congenitally abnormal as well as displaced.[3]

The entire group of anomalies would appear to be closely related in embryologic development, arising as the result of defective formation and differentiation of the ventral mesoderm at about 14–18 days of embryonic life.[1] On the basis of embryological development, this syndrome may be classified into two groups.[1] The first group arises as the result of developmental failure of a segment of the mesoderm and comprises three of the defects, i.e., diaphragmatic defect (which results from total or partial failure of the transverse septum to develop); pericardial defect (which is closely related to faulty development of the transverse septum); and intracardiac lesions (which is the result of faulty development of the epimyocardium, which is derived from the splanchnic mesoderm). The second group includes the sternal and abdominal wall defect and appears to arise due to failure of migration of the paired primordial structures.[1] Many variants of Cantrell's pentalogy have been described according to the postulated embryological development of these defects[1,4,5]; these various types may be classified as follows[2]:

**Class 1:** Exact diagnosis, with the five defects present

**Class 2:** Probable diagnosis, with four defects (including intracardiac and abdominal wall defects) present

**Class 3:** Incomplete diagnosis, with combination in the defects (always accompanied by sternal defects)

The occurrence of congenital intracardiac anomalies is a constant element of this syndrome; an interventricular septal defect was present in every instance in which a description of the heart was available, and was also found in our case.[1,3] Other intracardiac anomalies that are seen include interatrial septal defect (53%), valvular or infundibular pulmonary stenosis (33%), tetralogy of Fallot (20%), left ventricular diverticulum (20%), etc.[1] With the increasing use of antenatal diagnostic tools, these anomalies can be diagnosed before birth.[2,3] Treatment should consist of immediate surgical repair (except for the intracardiac abnormalities); some cases that had successful surgical correction have been mentioned in literature.[1,3–5] The prognosis in cases of ectopia cordis is much worse, with cases of thoracoabdominal ectopia showing slightly better prognosis than the other ectopias.[3]

Although most cases of the ectopia cordis appear as isolated, sporadic defects, other associated anomalies, including chromosomal abnormalities (trisomy 18), are reported in literature.[6] Carmi *et al.*[7] described cleft lip, with or without cleft palate, and encephalocele associated with ventral midline anomalies within the spectrum of Cantrell's pentalogy as a subunit of the midline developmental anomalies.[7] In our case, cytogenetic analysis was not performed and there was no associated anomaly other than Cantrell's pentalogy.

In our case, the neonate had thoracoabdominal ectopia cordis with Cantrell's pentalogy and, therefore, he belongs to class 1 of the embryological classification. In developing countries like ours, there is a lack of adequate health services in remote rural areas. In the absence of antenatal diagnosis, the neonate was delivered at home without qualified medical assistance. He presented late with severe respiratory acidosis and dyselectrolytemia which resulted in the fatal outcome. Ghidini *et al.*[8] described 10 cases of prenatally diagnosed Cantrell's pentalogy, with a uniform fatal outcome.[8] To conclude, Cantrell's pentalogy with ectopia cordis is a lethal anomaly and prompt medical and surgical interventions are required immediately after birth.
